# The Garden at My Door

**Published:** 1893-05

**Authors:** Louise M. Fuller

**Affiliations:** Jacksonville, Ill.


### THE GARDEN AT MY DOOR.

Many northward now are faring,
With their health in anxious caring,
And others, many, trail from shore to shore.
All seem merely birds of passage ;—
I must wait a homely message,
And a journey in the garden at my door.

My door opens right upon it ;—
I run out without my bonnet
To watch my precious seedlings break the soil.
And delight in my own digging,
In some handy, cast-off rigging,
That neither sun, nor rain, nor mud can spoil.

Only see, how very charming
Are the strawberries in forming,
As I wait for them to ripen at my door.
Always something for a friend
And no hurts but I can mend
With the balsam that's growing at my door.

And every morning early,
While the dew is beaded, pearly,
I must see what the new day is bringing on.
And in the evening, weary,
There is nothing half so cheery
As a friendly eye, my garden beds upon.

With my tools and tired toiling,
What a deal of blessed moiling,—
The angel sleep no longer far away ;
And the birds they set me singing,
As around me they are winging,
While I'm weeding out my garden in the May.

Jacksonville, Ill. LOUISE M. FULLER.

